# Recent advances in the application of microbial diamine oxidases and other histamine-oxidizing enzymes

**DOI:** 10.1007/s11274-022-03421-2

**Published:** 2022-10-08

**Authors:** Lucas Kettner, Ines Seitl, Lutz Fischer

**Affiliations:** Department of Biotechnology and Enzyme Science, Institute of Food Science and Biotechnology, University of Hohenheim, Garbenstr. 25, 70599 Stuttgart, Germany

**Keywords:** Biogenic amines, Diamine oxidase, Histamine, Histamine intolerance, Histamine oxidizing enzymes

## Abstract

The consumption of foods fraught with histamine can lead to various allergy-like symptoms if the histamine is not sufficiently degraded in the human body. The degradation occurs primarily in the small intestine, naturally catalyzed by the human diamine oxidase (DAO). An inherent or acquired deficiency in human DAO function causes the accumulation of histamine and subsequent intrusion of histamine into the bloodstream. The histamine exerts its effects acting on different histamine receptors all over the body but also directly in the intestinal lumen. The inability to degrade sufficient amounts of dietary histamine is known as the ‘histamine intolerance’. It would be preferable to solve this problem initially by the production of histamine-free or -reduced foods and by the oral supplementation of exogenous DAO supporting the human DAO in the small intestine. For the latter, DAOs from mammalian, herbal and microbial sources may be applicable. Microbial DAOs seem to be the most promising choice due to their possibility of an efficient biotechnological production in suitable microbial hosts. However, their biochemical properties, such as activity and stability under process conditions and substrate selectivity, play important roles for their successful application. This review deals with the advances and challenges of DAOs and other histamine-oxidizing enzymes for their potential application as processing aids for the production of histamine-reduced foods or as orally administered adjuvants to humans who have been eating food fraught with histamine.

## Introduction

**Fig. 1:**
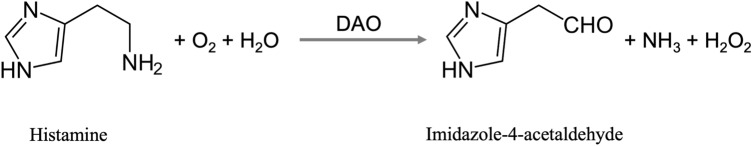
Oxidative deamination of histamine by DAO

The biogenic amine histamine is a molecule of high physiological importance as it is an important neurotransmitter and immunomodulator in the human body (Maintz and Novak 2007; Comas-Basté et al. 2020). However, histamine also exists in various foods as an undesired constituent (Karovičová and Kohajdová 2005). This is due to the presence of L-histidine decarboxylase (EC 4.1.1.22) that generates histamine from the respective precursor amino acid L-histidine through decarboxylation (Bloch and Pinösch 1936). The decarboxylase either endogenously exists in the raw food itself or, more relevantly, is produced by microbial contaminants during the processing, ripening or storage of the food (Karovičová and Kohajdová 2005). Histamine has received particular attention due to various outbreaks of food poisoning and for being the trigger of the condition ‘histamine intolerance’ (European Food Safety Authority 2011; Comas-Basté et al. 2020). Histamine can be found especially in fermented foods, such as cheese, sausage or sauerkraut, but is also found in non-fermented foods, such as microbially spoiled meat or fish, (Jarisch 2013; Santos 1996). Lactic acid bacteria especially are considered to be responsible for the formation of histamine in fermented foods (Spano et al. 2010). Since histamine is a molecule with various natural physiological functions in the human body, the consumption of dietary histamine can cause a multitude of different adverse physiological reactions if it is not efficiently degraded. The diversity in symptoms results from the activation of different histamine receptors (HR1, HR2, HR3 and HR4) in different cells all over the body like for example in the intestine (Jutel et al. 2009). Dale and Laidlaw (1910) showed that the administration of excessive histamine amounts can induce serious anaphylactic reactions in the mammalian body. This intoxication depends highly on the total amount of histamine consumed (European Food Safety Authority 2011). Even small amounts of dietary histamine, that would normally not cause any reaction, can induce allergy-like reactions in some susceptible people (European Food Safety Authority 2011). It is discussed that around 1% of the total population might be affected by this multifaceted condition known as ‘histamine intolerance’ (Jarisch 2013). Typical symptoms are the flushing and itching of the body, vomiting, diarrhea, abdominal pain and adverse effects on the cardiovascular system, such as hypotension, dizziness or tachycardia (Reese et al. 2021). This intolerance seems to derive from an impairment in the available activity of the histamine-degrading enzyme diamine oxidase (DAO, EC 1.4.3.22) (Gludovacz et al. 2016). Diamine oxidase belongs to the enzyme class of oxidoreductases and catalyzes the oxidative deamination of preferably diamines, some primary amines and rather fewer secondary and tertiary amines (McDonald et al. 2009; Mcgrath et al. 2009; Schwelberger and Bodner 1997). When histamine is the substrate, imidazole-4-acetaldehyde, ammonia and hydrogen peroxide are formed in the DAO-catalyzed reaction (Hrubisko et al. 2021) (Fig. 1).

Diamine oxidase is expressed especially in the intestine, kidney and placenta and stored in vesicular structures for secretion (Schwelberger et al. 1998; McGrath et al. 2009). This expression and storage of DAO in villus epithelial cells in the intestine represents the body’s first barrier against dietary histamine (Schwelberger et al. 1998). However, if the intestinal DAO activity available is not sufficient for the degradation of the particular amount of histamine, it can surpass into the bloodstream causing histamine-related symptoms. This DAO insufficiency might be due to either a genetic predisposition or be an acquired condition (Ayuso et al. 2007). The latter can be of a temporary nature and caused, for example, by DAO inhibition through certain types of medicine or by some gastrointestinal medical conditions (Schmidt et al. 1990; Leitner et al. 2014). Histamine intolerance cannot currently be treated with a specific medication. People who are affected can only prevent or reduce the symptoms occurring, sticking to a low-histamine diet (Reese et al. 2021). Commercially available dietary supplements, such as Daosin or different products from the company DR Healthcare, contain a protein extract from pig kidney and, as claimed by the manufacturers, are intended to support the endogenous DAO in the small intestine by delivering additional pig DAO. In fact, this idea of supplementing exogenous DAO to treat histamine intolerance symptoms was already implemented in 1936, when the company Bayer I.G. Farbenindustrie Aktiengesellschaft Leverkusen launched the product Torantil, that also contained a protein extract but from pig intestine (Meyer 2004).

However, Torantil was taken off the market in 1967 due to a lack of pharmacokinetic effectiveness (Meyer 2004). Several clinical studies have investigated the dietary supplements currently available for the treatment of histamine-related adverse physiological reactions and found that their administration led to symptom reductions (Komericki et al. 2011; Manzotti et al. 2016; Yacoub et al. 2018; Izquierdo-Casas et al. 2019; Schnedl et al. 2019). In contrast to this, it was shown recently that a commercially available preparation did not possess any DAO activity and that high DAO activities of at least 50 nkat are required to degrade food-relevant amounts of histamine in a buffered system (Kettner et al. 2020). Thereby, one nkat was defined as the amount of enzyme that converts 1 nmol histamine per second at 37 °C, which corresponds to 0.06 Enzyme Units (µmol histamine per minute). Furthermore, much higher activities of at least 690 nkat might be required when used under actual simulated intestinal conditions (Kettner et al. 2022). To obtain this DAO activity, around 1.4 kg pig kidneys would be required for the extraction and partial purification (Kettner et al. 2020). Conclusively, the extraction of DAO from pig kidney does not yield sufficient DAO activity for an economic application as dietary supplement.

The application of pig DAO for the reduction of histamine in foods is also not reasonable due to the high activity required. Naila et al. (2011, 2015) used a DAO preparation from pig liver and applied an activity of 42 µkat/L for the histamine degradation in tuna soup and found it to be useful for the degradation of relevant amounts of histamine. To put this into perspective, the DAO extraction and partial purification from around 80 kg pig kidneys would be necessary to obtain the DAO amount required for 1 L of this tuna soup (Kettner et al. 2020). In contrast, this DAO activity is obtainable by the disruption and purification of around 2 kg wet yeast mass of a genetically modified *Yarrowia lipolytica* PO1f strain that produces a microbial DAO (Kettner et al. 2022). However, the activity of 42 µkat/L used by Naila et al. is not an appropriate amount at all when a true application in industry would be considered.

The human DAO has a high affinity towards histamine with a *K*_m_ value of 0.0028 mM, which is a necessity to sufficiently regulate the low histamine concentration in the circulation (Elmore et al. 2002). Here, a plasma histamine concentration of 0.1 mg per liter was considered to be a concentration that can induce severe anaphylactic reactions (Boehm et al. 2019).

On the other hand, microbial histamine oxidizing enzymes (HOX) primarily seem to serve for the nitrogen provision of the cell and therefore have distinctively higher *K*_m_ values. Since the application of DAO in foods or as a dietary adjuvant for the histamine degradation in the intestine brings along much higher histamine concentrations than in plasma, the kinetics of the microbial HOX should be sufficient. Furthermore, the decreased activity at lower histamine concentrations can be compensated by the administration of higher enzyme amounts. In conclusion, the production of alternative HOX in microbial expression hosts will deliver sufficient enzyme activity for the production of effective dietary supplements or histamine-reduced foods, respectively.

## Classification of histamine-degrading enzymes

Regarding the evaluation of histamine-degrading enzymes, it has to be considered that not only DAOs (EC 1.4.3.22) are capable of converting histamine as the substrate. Primary amine oxidases (EC 1.4.3.21) and monoamine oxidases (EC 1.4.3.4) can also catalyze an oxidative deamination of histamine (Ochiai et al. 2006; Sugawara et al. 2015). According to the Nomenclature Committee of the International Union of Biochemistry and Molecular Biology (NC-IUBMB), the three enzyme classes are differentiated mainly based on their substrate preference and inhibiting compounds (McDonald et al. 2009). Unlike DAOs that preferably oxidize diamines, primary amine oxidases rather oxidize primary monoamines but show little or no activity towards diamines, and secondary and tertiary amines. Monoamine oxidases catalyze the oxidative deamination of primary amines and also some secondary and tertiary amines.

DAOs and primary amine oxidases employ trihydroxyphenylalanine quinone (TPQ) and metal ions like copper and calcium as cofactors (Mcgrath et al. 2009). On the other hand, monoamine oxidases comprise a covalently bound flavin adenine dinucleotide (FAD) as cofactor (Son et al. 2008).

The DAOs and primary amine oxidases are both inhibited by semicarbazide, which reacts with the carbonyl group of the TPQ cofactor. In contrast, monoamine oxidases are not inhibited by semicarbazide but by acetylenic compounds, such as chlorgyline, 1-deprenyl and pargyline. Besides these three enzyme classes, there are other, apparently less relevant enzyme classes, which also act on histamine (Table 1).

**Table 1:** Enzymes that metabolize histamine. The EC number, the catalyzed reaction and the cosubstrates are indicated

Recommended name	EC No.	Catalyzed reaction	Cosubstrate	References
Monoamine oxidase	1.4.3.4	Oxidative deamination	O_2_ H_2_O	Ochiai et al. (2006)
Putrescine oxidase	1.4.3.10	Oxidative deamination	O_2_ H_2_O	Bóka et al. (2012)
Protein-lysine 6-oxidase	1.4.3.13	Oxidative deamination	O_2_ H_2_O	Bollinger et al. (2005)
Primary amine oxidase	1.4.3.21	Oxidative deamination	O_2_ H_2_O	Sugawara et al. (2015)
Diamine oxidase	1.4.3.22	Oxidative deamination	O_2_ H_2_O	Schwelberger and Bodner (1997)
Methylamine dehydrogenase	1.4.9.1	Oxidative deamination	Amicyanin H_2_O	Bao et al. (2002)
Aralkylamine dehydrogenase	1.4.9.2	Oxidative deamination	Azurin H_2_O	Kondo et al. (2004)
L-cysteinyl-L-histidinylsulfoxide synthase	1.14.99.52	C-S bond formation	L-cysteine O_2_	Mashabela and Seebeck (2013)
Histamine N-methyltransferase	2.1.1.8	Methylation	S-adenosyl-L-methionine	Rajtar and Irman-Florjanc (2007)
Diamine N-acetyltransferase	2.3.1.57	N-acetylation	Acetyl-CoA	Wittich and Walter (1990)
Aralkylamine N-acetyltransferase	2.3.1.87	N-acetylation	Acetyl-CoA	Pan et al. (2018)
Gamma-glutamylhistamine synthethase	6.3.2.18	Peptide bond formation between histamine and L-glutamate	ATP L-glutamate	Stein and Weinreich (1982)

Histamine can be enzymatically modified in many different ways. However, as microbial alternatives to the mammalian DAO were found, this review deals with the enzymes catalyzing the oxidative deamination of histamine. Enzymes that metabolize histamine via a non-oxidative mechanism and EC 1.4.9.1, EC 1.4.9.2 and EC 1.14.99.52 require further cosubstrates that might not be present in the surrounding of the intended application and are therefore excluded for these considerations.

Therefore, enzyme classes, such as primary amine oxidases or monoamine oxidases, should also be considered when seeking alternatives to the DAO preparations currently available. These enzymes are mostly given trivial names in literature, for example, ‘histamine oxidase’ (Sekiguchi et al. 2004). These trivial names are not in accordance with the official EC nomenclature and make it challenging to classify these enzymes (Table 2).

**Table 2:** Histamine-oxidizing enzymes (HOX) described in literature. Their origin, trivial names, protein ID (Genbank), molecular weight (monomeric (M), dimeric (D) or tetrameric (T) enzyme) and the kinetic parameter *K*_m_ with histamine as the substrate are reported

Origin	Trivial name	Protein ID	Molecular weight [kDa]	*K*_m_ [mM]	Literature
*Arthrobacter aurescens* TC1	Amine oxidase (AMAO2)	ABM10002	72 ^+^	0.41	Lee and Kim (2013a)
*Arthrobacter aurescens* TC1	Amine oxidase (AMAO3)	WP_011777152	72 ^+^	0.88	Lee and Kim (2013a)
*Aspergillus carbonarius* AIU 205	Amine oxidase (I)	*	150 (D)	*	Sugawara et al. (2014)
*Aspergillus carbonarius* AIU 205	Amine oxidase (II)	OOF92112	130 (D)	*	Sugawara et al. (2015)
*Aspergillus carbonarius* AIU 205	Amine oxidase (III)	OOF94176	65 (M)	*	Sugawara et al. (2015)
*Arthrobacter crystallopoietes* KAIT-B-007	Histamine oxidase	BAE48148	81 (M)	0.51	Sekiguchi et al. ( 2004)
*Arthrobacter globiformis* IFO12137	Histamine oxidase	Q59118.3	75 (D)	0.06	Choi et al. (1995)
*Arthrobacter globiformis* IFO12137	Phenylethylamine oxidase	WP_003799421	141 (D)	*	Tanizawa et al. (1994)
*Aspergillus niger* AKU 3302	Amine oxidase (AO-I)	Q12556	150 (D)	*	Frébort et al. (1996)
*Aspergillus niger* AKU 3302	Amine oxidase (AO-II)	*	80 (M)	*	Frébort et al. (1996)
*Glutamicibacter* sp. N1A3101	Histamine oxidase	QXO85771	*	*	Sadeghi et al. (2020)
*Klebsiella aerogenes* W70	Monoamine oxidase	P49250	79 (M)	*	Yamashita et al. (1993)
*Kluyveromyces marxianus* CBS 5795	Amine oxidase	KAG0676903	150 (D)	0.2	Corpillo et al. (2003)
*Lathyrus sativus* (pea)	Diamine oxidase	Q6A174	148 (D)	0.11	Fusco et al. (2011) Šebela et al. (1998)
*Mycobacterium* sp. strain JC1	Amine oxidase	ACS29498	150 (D) 300 (T)	*	Lee et al. (2008)
Pig	Diamine oxidase	Q9TRC7	186 (D)	0.02	Schwelberger and Bodner (1997)
*Yarrowia lipolytica* PO1f	Diamine oxidase	Q6CGT2	75 (D)	2.3	Kettner et al. (2021)

*No information available in the respective literature source

^+^Theoretical molecular weight calculated from amino acid sequence. No data available on the number of enzyme subunits

**Fig. 2:**
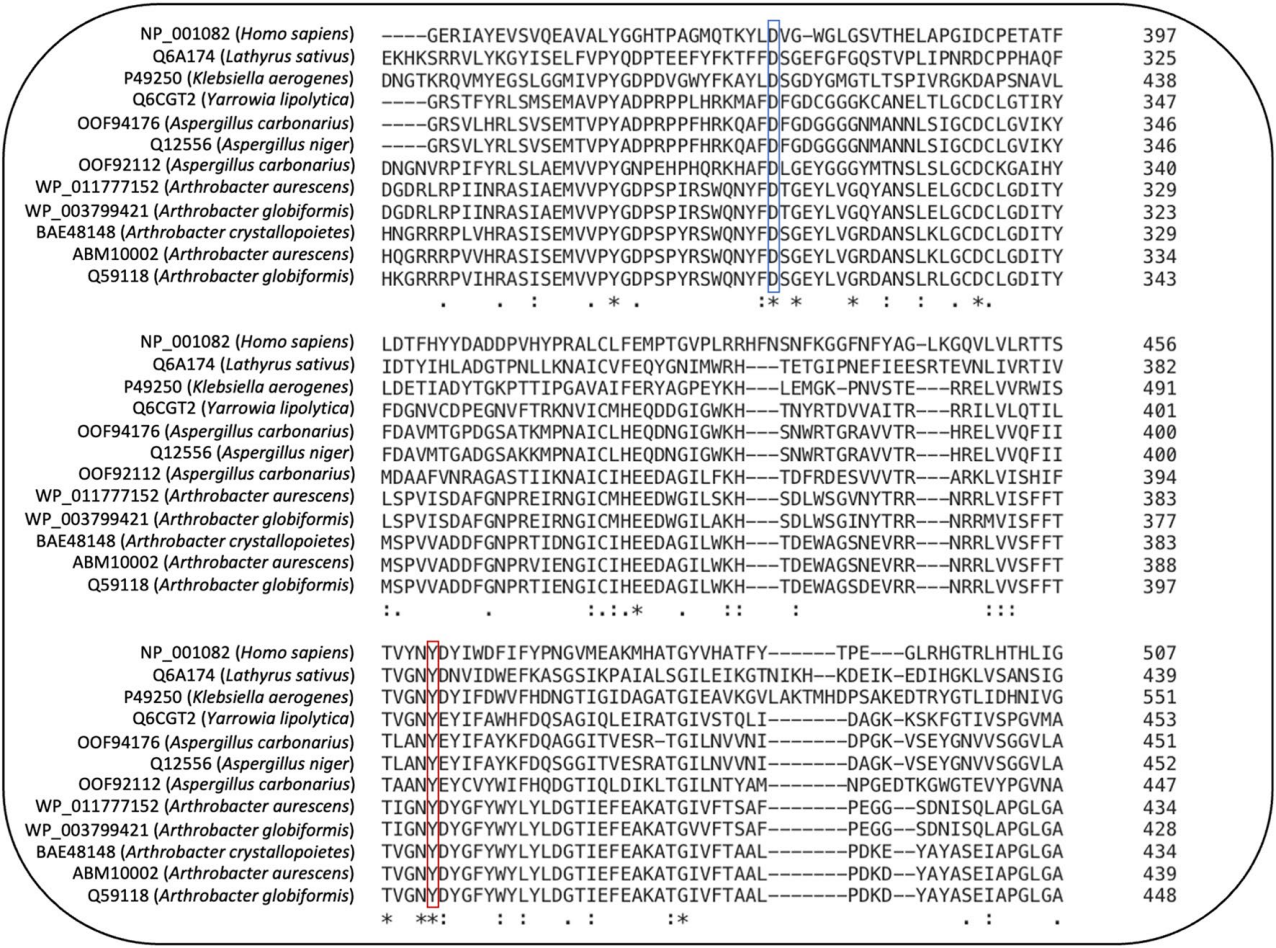
Partial amino acid alignment of microbial HOX and comparison with the *Homo sapiens* (human) and *Lathyrus sativus* (pea) DAO. Blue and red framing indicate active sites (373 (D) = aspartic acid and 461 (Y) = tyrosine) of the human DAO. * = fully conserved residue; : = conservation between groups of strongly similar properties; . = conservation between groups of weakly similar properties. Created with Clustal Omega (Sievers et al. 2011)

The amino acid sequences of the different HOX are very different when compared to each other (Fig. 2). Nevertheless, all of them share the same characteristic active site residues that are also found in human DAO. These are an aspartic acid at position 373 and a tyrosine at position 461 in the human DAO amino acid sequence (McGrath et al. 2009). The protein-derived TPQ cofactor of DAO is formed posttranslationally from Tyr461 in the presence of oxygen and copper ions and seems to be present in all microbial HOX found in literature and also in a vegetal DAO from *Lathyrus sativus* (McGrath et al. 2009) (Fig. 2).

Furthermore, other residues, for example, Tyr463 or N460, are also highly conserved in HOX. However, the catalytic role of these is not currently described in literature.

## Substrate selectivity

**Fig. 3:**
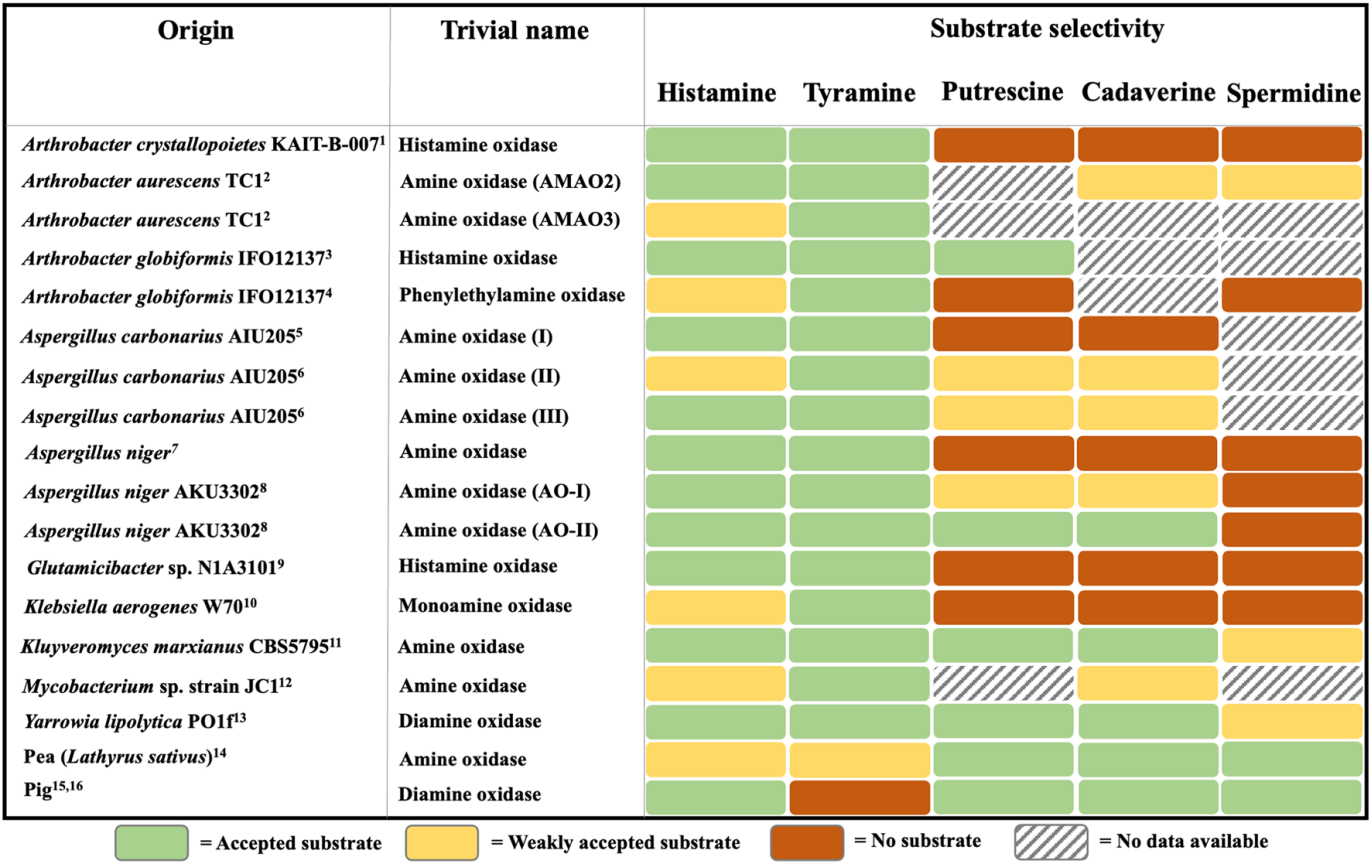
Substrate selectivity of microbial, vegetal (*Lathyrus sativus*) and animal (pig) HOX found in literature. ‘Weakly accepted substrate’ (yellow) refers to an enzyme activity of less than 8% compared to the most favored substrate or if the activity/selectivity was described as “weak” in the respective literature. ^1^(Sekiguchi et al. 2004), ^2^(Lee and Kim 2013a), ^3^(Choi et al. 1995),^4^(Shimizu et al. 1997), ^5^(Sugawara et al. 2014), ^6^(Sugawara et al. 2015), ^7^(Schilling and Lerch 1995), ^8^(Frébort et al. 1996), ^9^(Sadeghi et al. 2020), ^10^(Yamashita et al. 1993), ^11^(Corpillo et al. 2003),^12^(Lee et al. 2008), ^13^(Kettner et al. 2021), ^14^(Šebela et al. 1998), ^15^(Schwelberger and Bodner 1997), ^16^(Hill et al. 1970)

In addition to histamine, other biogenic amines, such as tyramine, putrescine or cadaverine, are also frequently found in foods and can cause toxicological effects in the human body (Ladero et al. 2010; del Rio et al. 2019). These are also formed in foods due to the presence of L-amino acid decarboxylases (Huang et al. 2018). The mammalian DAOs from human and pig kidney oxidatively deaminate histamine, putrescine and cadaverine (Schwelberger and Bodner 1997; Elmore et al. 2002). However, tyramine has been reported not to be oxidized by pig kidney DAO (Hill et al. 1970). A broad substrate selectivity by microbial HOX is desired because histamine is not the only biogenic amine that can be found in food. The microbial HOX described in literature present a diverse substrate selectivity (Fig. 3).

**Fig. 4:**
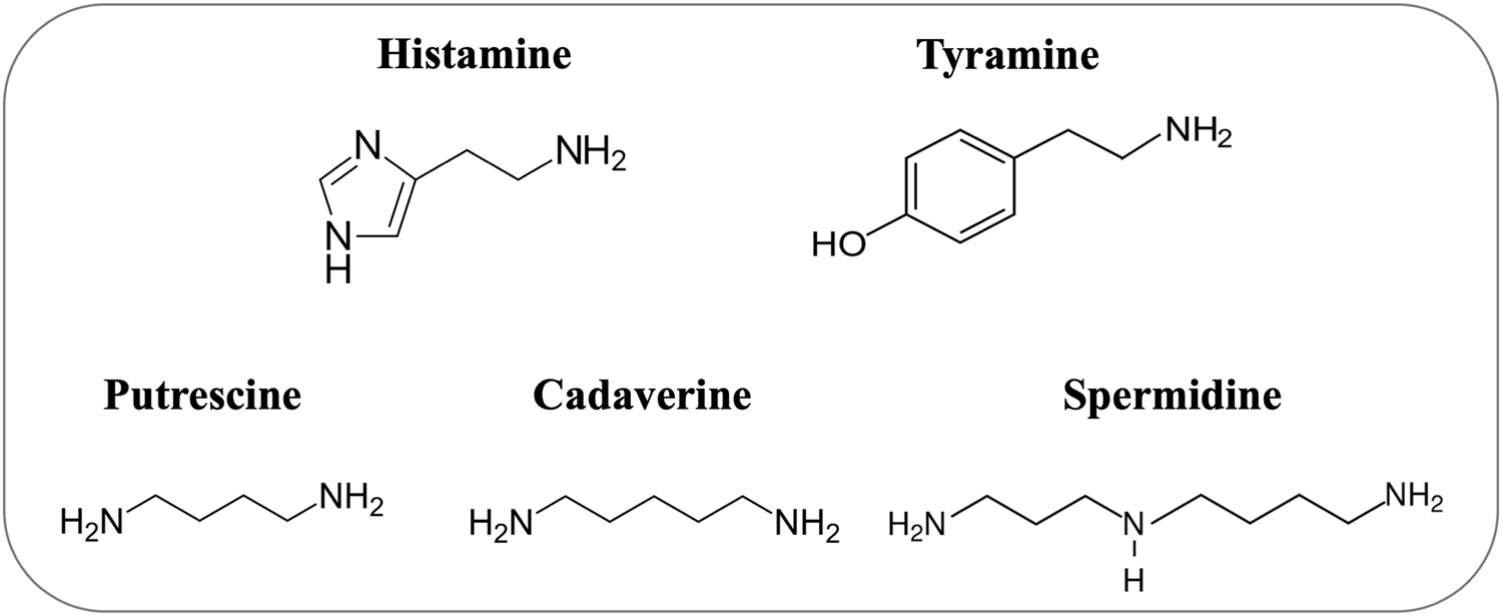
Structural formulae of the biogenic amines histamine, tyramine, putrescine, cadaverine and spermidine

Interestingly, all of the microbial HOX found in literature also oxidatively deaminate tyramine. This might be due to the similarity in the distance of the aromatic ring system to the primary amine, which is rather different to the other accepted biogenic amines (Fig. 4).

If the HOX is also thought to be used for the degradation of other relevant biogenic amines, such as tyramine, cadaverine and putrescine, only the ‘amine oxidases’ from *A. carbonarius* AIU205, *K. marxianus* CBS5795 and *A. niger* AKU3302 as well as the DAO from *Y. lipolytica* PO1f would be suitable (Frébort et al. 1996; Corpillo et al. 2003; Sugawara et al. 2015; Kettner et al. 2021). Hereby, the ‘amine oxidase’ from *K. marxianus* CBS5795 and the DAO from *Y. lipolytica* PO1f showed the broadest substrate selectivity since they also deaminated spermidine oxidatively.

## Effect of the surrounding pH-value on enzyme stability and activity

Microbial HOX could be applicable as orally administered tablets for the degradation of histamine in the human small intestine and processing aids for the preparation of histamine-reduced foods. In the respective surrounding, the resulting aldehyde and hydrogen peroxide reaction products should be metabolized to further breakdown products and therefore not be of concern. A gastric acid resistant capsule preparation should be chosen in order to transport the active HOX to the small intestine when applied in humans as an orally administered tablet. Thus, only the intestinal conditions are of relevance for the enzymes’ properties. The United States Pharmacopeia specifies a neutral pH of 6.8 for a simulated intestinal fluid (United States Pharmacopeia 2022). The latter contains monobasic potassium phosphate, sodium hydroxide and the enzyme preparation ‘pancreatin.’ Pancreatin is a mixture of different enzyme activities, such as amylases, lipases and peptidases (Salhi et al. 2020). The latter cause a rapid degradation of microbial and animal HOX, which further highlights the need for the administration of high activities to compensate for activity losses (Kettner et al. 2020, 2022). The half-life period of free pig DAO in a simulated intestinal fluid was 19 min (Kettner et al. 2020). It was shown with a microbial DAO in a simulated intestinal fluid, that the amount and type of food consumed has a distinct effect on the stability of a supplemented DAO, extending its half-life period to at least 30 min (Kettner et al. 2022). In contrast, a vegetal DAO from pea (*L. sativus*) showed a high stability in a simulated intestinal fluid with a half-life period of around 16 h (Blemur et al. 2016).

If HOX are thought to be used as processing aids for the production of histamine-reduced foods, for example, in cheese or sausage, the individual composition of the food and the surrounding conditions during the food fermentation must be considered. These types of foods often exhibit an acidic pH-value due to the production of lactic acid by lactic acid bacteria that are added as starter cultures to the fermentation process. Lee and Styliadis (1996) investigated different salamis, sausages and ham and found pH-values between 4.3 and 6.4 in these foods. This pH-range could also be expected for ripened cheeses. Fröhlich-Wyder et al. (2015) measured a pH-value of 5.7 in a Tilsit-type cheese that had been aged for 90 days. In conclusion, the desirable microbial histamine-oxidizing enzymes should be stable and sufficiently active under neutral or slightly acidic conditions for the reduction of histamine under intestinal or food-relevant conditions, respectively.

**Fig. 5:**
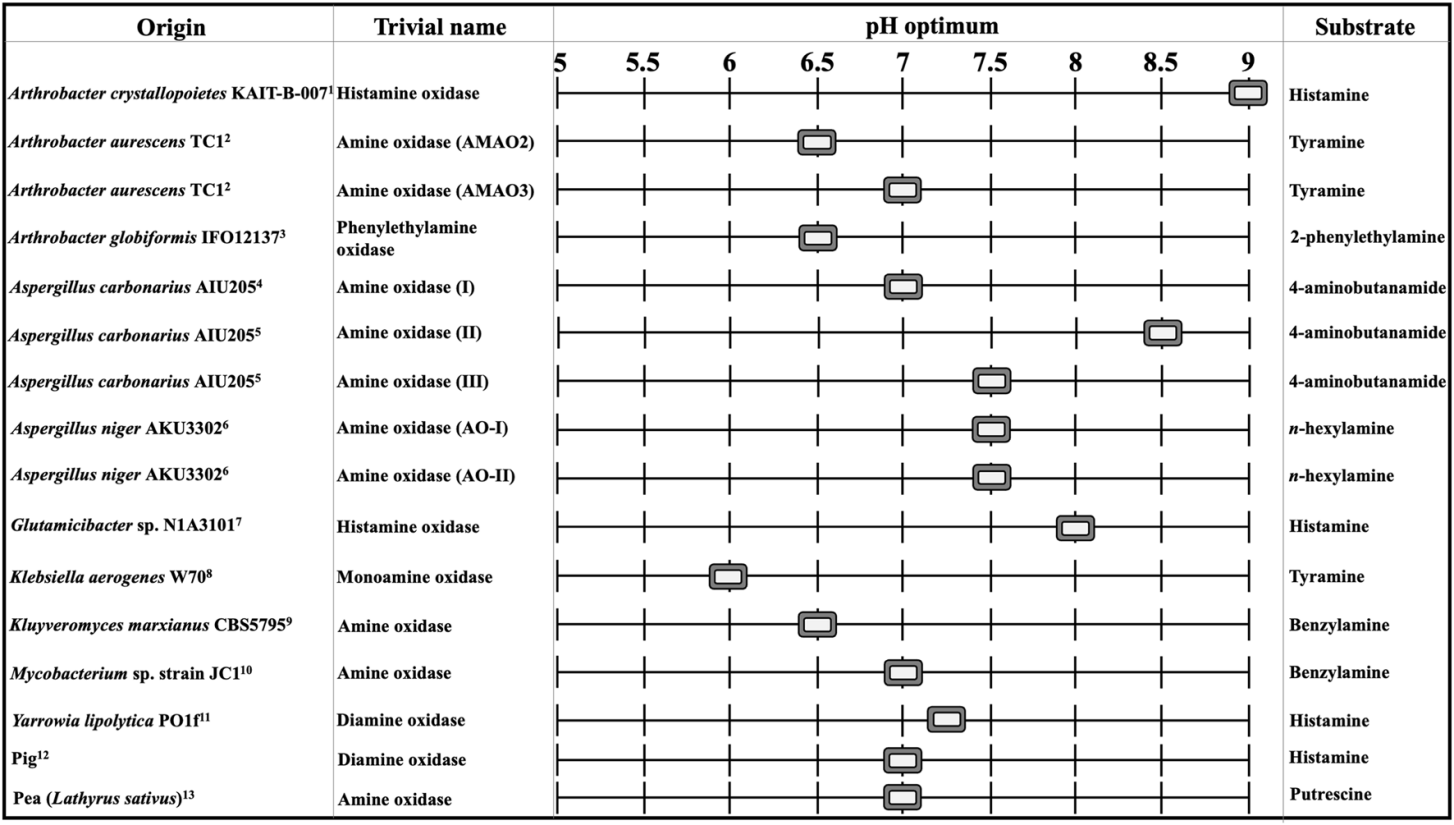
Microbial, pig and pea HOX, their trivial names as denoted in the respective literature, their optimum pH and the substrate used for the HOX activity determination. ^1^(Sekiguchi et al. 2004), ^2^(Lee and Kim 2013a), ^3^(Shimizu et al. 1997), ^4^(Sugawara et al. 2014), ^5^(Sugawara et al. 2015), ^6^(Frébort et al. 1996), ^7^(Sadeghi et al. 2020), ^8^(Yamashita et al. 1993), ^9^(Corpillo et al. 2003),^10^(Lee et al. 2008), ^11^(Kettner et al. 2021), ^12^(Mondovì et al. 1964), ^13^(Šebela et al. 1998)

Microbial HOX described in literature generally show maximal activity under neutral to slightly acidic or slightly basic conditions (Fig. 5).

It must be considered that the pH and temperature profiles for the different microbial HOX found in literature have been investigated with different analytical methods, substrates and in different buffer systems. The pH profiles were investigated with histamine as the substrate only for a ‘histamine oxidase’ from *Arthrobacter crystallopoietes* KAIT-B-007 and *Glutamicibacter* sp. N1A3101, and a DAO from *Yarrowia lipolytica* PO1f (Sekiguchi et al. 2004; Sadeghi et al. 2020; Kettner et al. 2021). The two ‘histamine oxidases’ and an ‘amine oxidase’ from *Aspergillus carbonarius* AIU205 showed maximal activity under basic conditions of pH 9, 8 and 8.5, respectively (Sekiguchi et al. 2004; Sugawara et al. 2015; Sadeghi et al. 2020). These enzymes might be inadequate for application in the human intestine or fermented foods if the activity decreases rapidly under neutral or slightly acidic conditions. On the other hand, a monoamine oxidase from *Klebsiella aerogenes* W70 showed maximal activity at pH 6 with tyramine as the substrate and was reported to be stable until pH 4. If the same pH profile was obtained with histamine as the substrate, the latter could be a useful HOX for the histamine degradation in fermented foods. However, the other microbial HOX might also be applicable when adequate activities are used to compensate for the decreased activity under suboptimal conditions. The DAO from *Y. lipolytica* PO1f, for example, showed around 50% of its activity at pH 6.2 when compared to its maximal activity at pH 7.2, which could be compensated by the application of twice the amount of enzyme for the histamine degradation in a slightly acidic environment. Microbial HOX are generally most active and stable in a neutral pH environment. This is conclusive because these enzymes seem to be naturally located inside the cell, where a neutral pH generally exists. It would be very interesting to discover an extracellular HOX because it is known that secreted enzymes are more stable and could be more active under acidic conditions.

## Effect of the surrounding temperature on enzyme stability and activity

The HOX should be sufficiently active and stable at 37 °C if thought to be administered as a dietary adjuvant to degrade histamine in the human intestine.

**Fig. 6:**
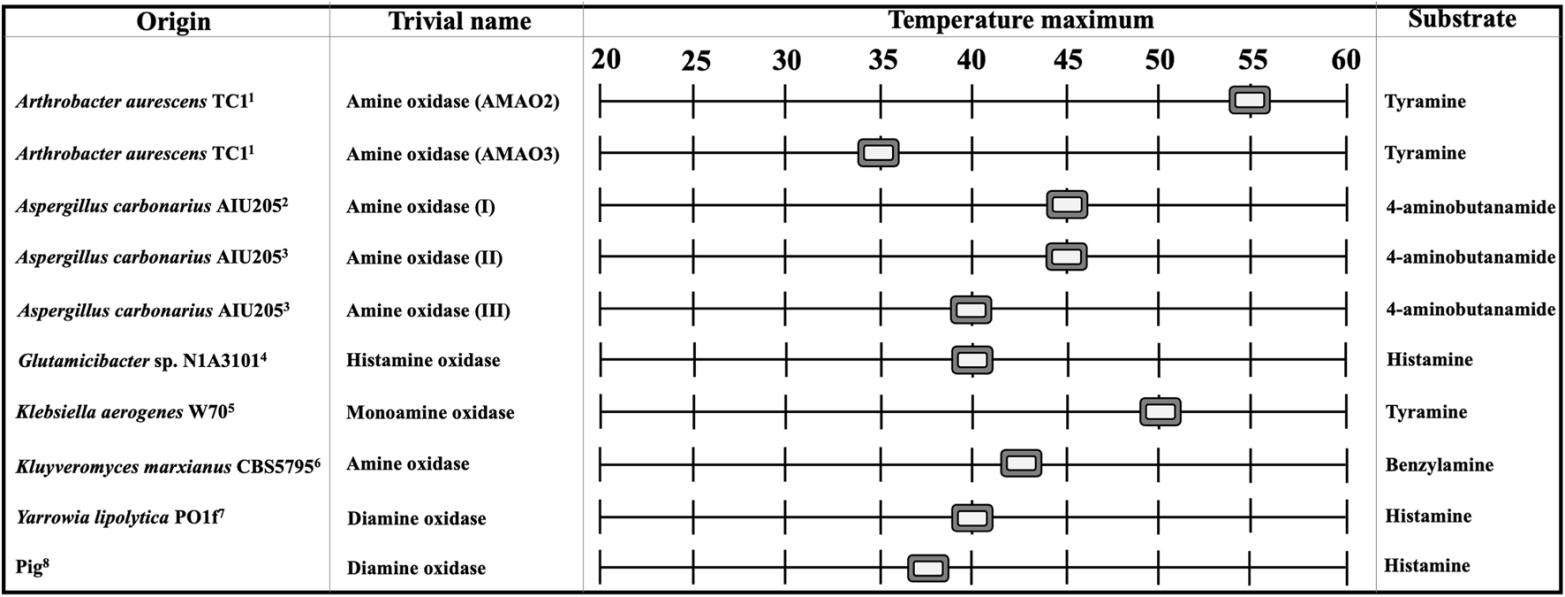
Microbial and pig HOX, their trivial names as denoted in the respective literature, their temperature maximum and the substrate used for the HOX activity determination. ^1^(Lee and Kim 2013a), ^2^(Sugawara et al. 2014), ^3^(Sugawara et al. 2015), ^4^(Sadeghi et al. 2020), ^5^(Yamashita et al. 1993), ^6^(Corpillo et al. 2003), ^7^(Kettner et al. 2021), ^8^Dapkevicius et al. (2000)

By contrast, when added to a food fermentation process as a processing aid, the HOX administered must be active at a distinctively lower process temperature. Exemplarily, the temperature often used for the ripening process in cheese production ranges between 5 and 20 °C (Terri and Boylston 2012). The temperature ranges between 6.5 and 18.3 °C for the ripening of a Sicilian salami in a traditional ripening room (Moretti et al. 2004). The HOX found in literature show mesophilic to thermophilic properties and are shown in Fig. 6.

An ‘amine oxidase’ from *A. aurescens* TC1 (AMAO2) and a monoamine oxidase from *K. aerogenes* W70 especially show maximal activity at 55 and 50 °C, respectively. Thereby, the ‘amine oxidase’ from *A. aurescens* TC1 (AMAO2) showed a rather broad temperature profile with an enzyme activity (towards tyramine) of around 30% at 10 °C when compared to its maximal activity at 55 °C (Lee and Kim 2013b). Hence, HOX of a thermophilic nature might also still be applicable for the histamine reduction in fermented foods if the temperature profile shows reasonable activity at lower temperatures and if administered in sufficient amounts.

All of the microbial HOX described in literature might be suitable for the histamine reduction under intestinal conditions regarding their temperature profiles. However, in addition to the enzyme activity, the thermal enzyme stability is also of high relevance. The HOX from the family of *Micrococcaceae*, such as *Arthrobacter* or *Glutamicibacter*, especially seem to have a remarkable thermostability when compared to other HOX. The ‘histamine oxidase’ from *A. crystallopoietes* KAIT-B-007 retained around 70% of its activity after an incubation at 70 °C for 60 min (Sekiguchi et al. 2004). However, the DAO from *Y. lipolytica* PO1f, which holds rather mesophilic characteristics, also retained around 90% of its activity after an incubation at 37 °C for 5 h (Kettner et al. 2021).

In conclusion, most of the HOX reported in literature seem to be applicable for the histamine reduction under intestinal conditions regarding their pH and temperature profiles. If used in fermented foods, where a more acidic and colder environment is generally present, the loss of activity and stability must be compensated by the application of higher amounts of HOX.

## Application of histamine-oxidizing enzymes for the histamine reduction in foods or as dietary adjuvants

The DAOs isolated from vegetal and animal sources have already been investigated for the histamine reduction in foods and the intestine (Naila et al. 2015; Kettner et al. 2020; Neree et al. 2020). The microbial HOX have not yet been comprehensively investigated for these applications. However, biogenic amines were reduced in some exemplary foods by inoculation with oxidase-positive microorganisms without an in-depth characterization of the enzymes responsible ( Naila et al. 2010). Leuschner and Hammes 1998, for example, used the bacterium *Brevibacterium linens* to prepare a Munster cheese with reduced histamine and tyramine contents. Furthermore, Bäumlisberger et al. (2015) found that the yeast *Debaryomyces hansenii* H525 was able to degrade histamine and tyramine in grape juice. The enzyme responsible for this degradation was extracted from *D. hansenii* H525 cells and used only in a synthetic buffer system to degrade the biogenic amines histamine and tyramine. However, the conversion of these biogenic amines in a true food matrix was not investigated. The application of HOX in whole-cell systems can be beneficial, especially for administration in fermented foods with pH conditions unsuitable for enzyme catalysis and stability. However, this coinoculation might also be problematic, causing a deterioration of the products’ sensorial properties and must be individually assessed (Bäumlisberger et al. 2015). Therefore, using free HOX with suitable biochemical properties for the intended application is the superior and better approach. However, the latter has not yet been shown in the literature and the question whether the application of microbial HOX for the histamine reduction in foods is possible remains unclear.

The application of microbial HOX as a dietary adjuvant for the histamine degradation in the human intestine was investigated with the DAO from *Y. lipolytica* PO1f (Kettner et al. 2022). Here, the DAO was prepared as a sucrose-based tablet and it reduced high amounts of histamine under simulated intestinal conditions. This showed that a microbial HOX could have the potential to help people with histamine intolerance. However, it was also found that very high enzyme activities are necessary to compensate for the losses through proteolytic digestion by the pancreas’ peptidases. This further highlights the need for a sufficient microbial production of the HOX desired.

## Microbial production of histamine-oxidizing enzymes

**Fig. 7:**
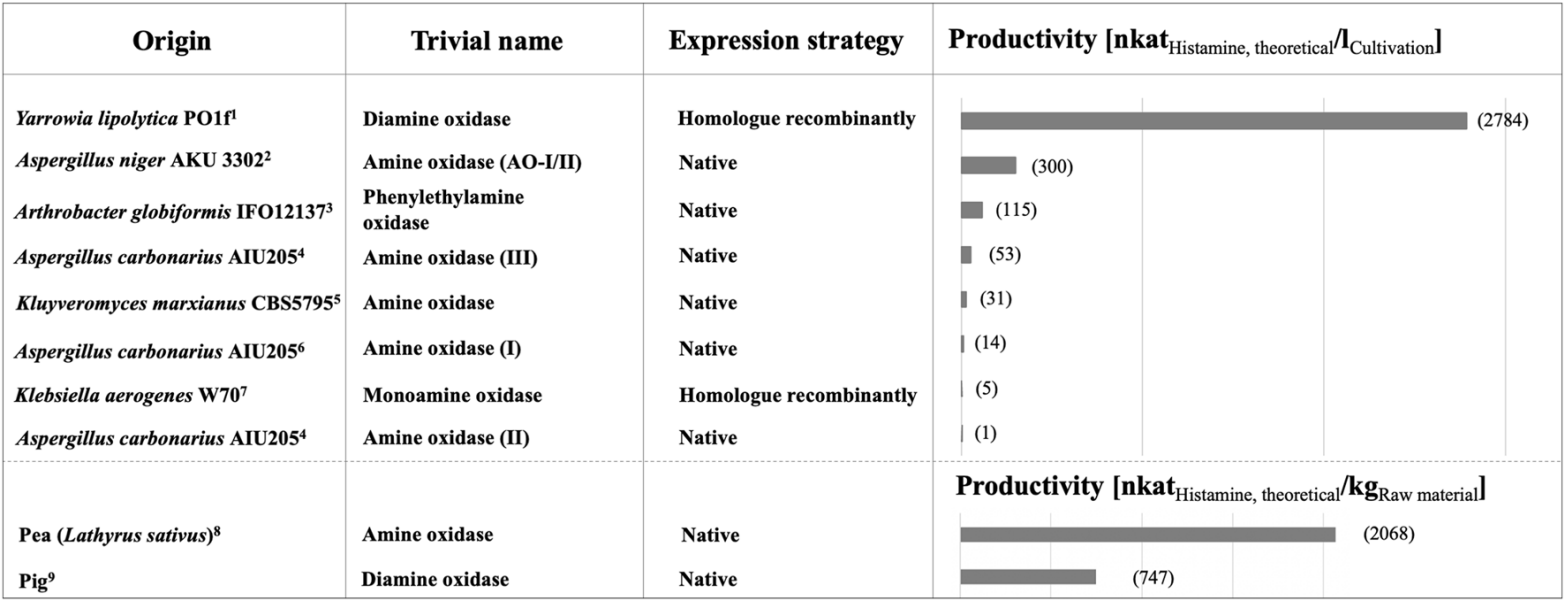
Theoretical productivities of microbial, vegetal (pea) and animal (pig) HOX (trivial names as denoted in the respective literature). Productivities are given as theoretical values if histamine was used as the substrate and are calculated from the kinetic data. ^1^(Kettner et al. 2021), ^2^(Frébort et al. 1996), ^3^(Shimizu et al. 1997), ^4^(Sugawara et al. 2015), ^5^(Corpillo et al. 2003), ^6^(Sugawara et al. 2014), ^7^(Yamashita et al. 1993), ^8^(Šebela et al. 1998), ^9^(Kettner et al. 2020)

In addition to the biochemical properties, also the economic producibility of each HOX are highly relevant for a successful application. Most of the HOX described in the literature were produced in their native expression hosts without any genetical modifications of the expression systems. This resulted in low activity yields when compared to modified expression systems in combination with the use of highly efficient promoters (Kettner et al. 2021). The productivities found in literature are challenging to compare due to the usage of different substrates, analytical methods and buffer systems for the HOX activity determinations. Nevertheless, an approximation can be given based on the kinetic data by calculating the theoretical productivity normalized for histamine as substrate (Fig. 7).

The productivities of the different HOX show that the recombinant production in a suitable and enhanced expression host outcompetes the native microbial, animal and vegetal production. The highest activity value (2784 nkat/l_Cultivation_) for the production of a HOX was obtained for a DAO from the yeast *Y. lipolytica* PO1f, that was produced homologously recombinantly under the control of the constitutive UAS1B8_TEF(136) promoter (Kettner et al. 2021). Here, the DAO gene was cloned into the genome of *Y. lipolytica* PO1f using the CRISPR-Cas9 system.

In addition, a monoamine oxidase from *K. aerogenes* W70 was produced homologously recombinantly, whereby lower activity values of 5 nkat/l_Cultivation_ were obtained (Yamashita et al. 1993). The monoamine oxidase was expressed using a plasmid-based expression strategy. The ‘amine oxidases’ from *A. aurescens* TC1 and a ‘histamine oxidase’ from *A. globiformis* IFO12137 were produced in *Escherichia coli* (Choi et al. 1995; Lee and Kim 2013a). However, the respective literature does not provide information to evaluate the producibility of each enzyme. Nevertheless, it can be concluded that the expression of HOX in *E. coli* might be challenging, as experienced by Choi et al. (1995) and Lee and Kim (2013a). Interestingly, the recombinant production of the *Y. lipolytica* DAO in *E. coli* Rosetta 2 (DE3) also resulted in low activity values of 66 nkat/l_Cultivation_ when compared to the homologous recombinant production (personal communication).

**Fig. 8:**
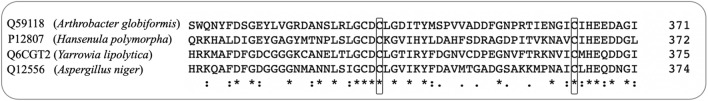
Partial alignment of the amino acid sequences of the HOX from *A. globiformis*, *H. polymorpha*, *Y. lipolytica* and *A. niger* highlighting conserved cysteine residues that might possibly be involved in the formation of a disulfide bond. * = fully conserved residue; : = conservation between groups of strongly similar properties;. = conservation between groups of weakly similar properties. Created with Clustal Omega (Sievers et al. 2011)

As discovered for the human DAO, disulfide bonds are relevant for its conformation, for example, by covalently linking the two subunits of the homodimeric enzyme (McGrath et al. 2009). Additionally, a ‘copper amine oxidase’ from *Hansenula polymorpha* was described to possess a disulfide bond that is relevant for its conformation, which seems to be a conserved motif in ‘copper amine oxidases’ (Li et al. 1998). The ‘histamine oxidase’ (Uniprot: Q59118) from *A. globiformis*, the ‘copper amine oxidase 1’ (Uniprot: Q12556) from *A. niger* and the DAO (Uniprot: Q6CGT2) from *Y. lipolytica* also seem to possess the conserved cysteine residues that are found in the amino acid sequence of *H. polymorpha* at the positions Cys338 and Cys364 (Fig. 8). This suggests that disulfide bonds might play a relevant structural role in HOX in general. Therefore, a suitable expression host which allows a correct enzyme assembly and disulfide bond formation is crucial for efficient production.

Although *E. coli* is often the first choice for heterologous enzyme expression, it is not always the best choice, especially for the expression of enzymes that require disulfide bond formation (Ke and Berkmen 2014). The expression of a protein in the cytoplasm of *E. coli*, which comprises a reducing environment, might lead to the expression of a misfolded protein (Derman and Beckwith 1991). Therefore, refolding of the latter or expression in a genetically optimized *E. coli* strain (such as Origami or SHuffle (de Marco 2009)), that facilitates disulfide bond formation, might be necessary (White et al. 1994). Using these *E. coli* hosts requires extensive optimization and might not be adequate to provide sufficient activity values. Thus, the expression of HOX in yeasts such as *Komagataella phaffii* should lead to high activity yields due to a more suitable cytoplasmic environment for correct protein folding and is recommended for further production studies (White et al. 1994).

## Histamine-oxidizing enzymes in biosensors

So far, microbial HOX have predominantly been thought to be used for analytical tasks, such as in a biosensor for the detection of biogenic amines (Lee and Kim 2013a; Sadeghi et al. 2020). For this purpose, DAO from pig and plant origin have already been extensively investigated (Apetrei and Apetrei 2016; Hernández-Cázares et al. 2011; Pérez et al. 2013). However, due to a broad substrate selectivity, the latter can only be applied for the detection of the total amount of biogenic amines and not specifically for histamine. The clear advantage of some microbial HOX for biosensor application is the high substrate specificity, allowing the development of a biosensor specifically for the detection of a particular biogenic amine. Bóka et al. (2012) developed a putrescine biosensor based on a putrescine oxidase from the bacterium *Kocuria rosea*. The putrescine oxidase showed high specificity towards putrescine and only minor towards cadaverine, tryptamine, spermidine, tyramine and histamine, and might, therefore, be useful for the detection of putrescine in food products. The ‘histamine oxidases’ from *A. crystallopoietes* KAIT-B-007 and *Glutamicibacter* sp. N1A3101 could have special potential for the development of a histamine biosensor because they show the highest activity towards histamine and no or little activity to the other common biogenic amines found in foods (Sadeghi et al. 2020; Sekiguchi et al. 2004).

## Conclusion

So far, microbial HOX have not yet been extensively investigated for their applicability as a dietary adjuvant or a processing aid to reduce histamine and other biogenic amines in the human intestine or fermented foods. The applicability for these purposes depends greatly on the enzymes’ biochemical properties. Since microbial HOX seem to be naturally expressed intracellularly, they are adapted to moderate surrounding conditions of about pH 7. Hence, showing the highest activity and stability in the neutral pH range and at moderate temperatures of around 30–45 °C. These properties make the microbial HOX capable of the degradation of histamine under intestinal conditions if delivered as a dietary adjuvant. The histamine reduction during a fermentation process of food with microbial HOX could be seen to be more difficult and should be further investigated to see whether losses of activity and stability in the acidic and colder surroundings can be compensated for by the addition of higher amounts of HOX.

An efficient microbial production of the enzyme desired is generally a prerequisite if HOX are intended to be used for these industrial processes. Thereby, the expression in yeasts, such as *Y. lipolytica* or *K. phaffii*, is preferable for this enzyme class. Furthermore, screening for new microbial HOX, especially of a psychrophilic nature, could deliver enzymes more suitable for application as processing aids in the fermentation of foods.
